# Experimental modelling of cardiac pressure overload hypertrophy: Modified technique for precise, reproducible, safe and easy aortic arch banding-debanding in mice

**DOI:** 10.1038/s41598-018-21548-x

**Published:** 2018-02-16

**Authors:** David Merino, Aritz Gil, Jenny Gómez, Luis Ruiz, Miguel Llano, Raquel García, María A. Hurlé, J. Francisco Nistal

**Affiliations:** 1 Hospital Universitario Marqués de Valdecilla, Avda. Valdecilla s/n, Santander, E-39008 Cantabria Spain; 2 Universidad de Cantabria, Facultad de Medicina, Santander, E-39011 Cantabria Spain; 3 Instituto de Investigación Valdecilla (IDIVAL), Cardenal Herrera Oria Av. s/n, Santander, E-39011 Cantabria Spain; 4 Centro de Investigación Biomédica en Red Cardiovascular (CIBERCV), Instituto de Salud Carlos III, Santander, Spain

## Abstract

Pressure overload left ventricular hypertrophy is a known precursor of heart failure with ominous prognosis. The development of experimental models that reproduce this phenomenon is instrumental for the advancement in our understanding of its pathophysiology. The gold standard of these models is the controlled constriction of the mid aortic arch in mice according to Rockman’s technique (RT). We developed a modified technique that allows individualized and fully controlled constriction of the aorta, improves efficiency and generates a reproducible stenosis that is technically easy to perform and release. An algorithm calculates, based on the echocardiographic arch diameter, the intended perimeter at the constriction, and a suture is prepared with two knots separated accordingly. The aorta is encircled twice with the suture and the loop is closed with a microclip under both knots. We performed controlled aortic constriction with Rockman’s and the double loop-clip (DLC) techniques in mice. DLC proved superiority in efficiency (mortality and invalid experiments) and more homogeneity of the results (transcoarctational gradients, LV mass, cardiomyocyte hypertrophy, gene expression) than RT. DLC technique optimizes animal use and generates a consistent and customized aortic constriction with homogeneous LV pressure overload morphofunctional, structural, and molecular features.

## Introduction

Pressure overload left ventricular hypertrophy (LVH) is shared by aortic valve stenosis, systemic hypertension and aortic coarctation and underlies several dire, well known, consequences in the long term evolution of these diseases^1–3^.

Animal models that reproduce LVH and its regression experimentally are instrumental for our understanding of the cellular and molecular mechanisms involved. The most popular of these models is the constriction of the mid transverse aortic arch (TAC), described by Rockman *et al*.^4^ who produced a gaged aortic stenosis laying a 27 G cannula parallel to the aortic arch, tying a ligature around both vessel and cannula, and then removing carefully the cannula. Theoretically, with this maneuver the residual aortic lumen would equal the cannula’s cross-section. The Rockman’s TAC technique (RT) produces a significant aortic gradient and a robust concentric LV hypertrophy in a few days. However, RT has several drawbacks. First and foremost, RT aims at inducing an identical flow area for any animal regardless of its habitus and, consequently, the degree of aortic constriction is inherently variable; the LV from a bigger mouse would face a greater pressure overload than a smaller animal. In addition, RT is highly operator-dependent, poorly reproducible, technically demanding and traumatic for the aorta.

We propose an alternative model which, instead of adjusting the luminal area of the constricted aorta to a particular value (defined by a reference mandrel), generates a fully controlled, predefined degree of stenosis dimensioned to the individual aortic arch diameter of each mouse. The method is also simpler and less traumatic to the aorta and allows a good standardization of the constriction irrespective of mouse’s weight, size or gender.

## Methods

The study was approved by the University of Cantabria Institutional Laboratory Animal Care and Use Committee and carried out in accordance with the Declaration of Helsinki and European Communities Council Directive (86/609/EEC). The chronogram of the different experimental series is depicted in Supplementary Figure 1. All the animals were operated, with either of the techniques, by the senior author (JFN) during the same time frame. Additional information in Supplementary Methods.

### Surgical technique

#### Rockman’s technique

Mice were anesthetized with intraperitoneal ketamine (100 mg/kg) and xylazine (5 mg/kg) and fixed in supine position on a servo-controlled heating pad.

**Figure 1:**
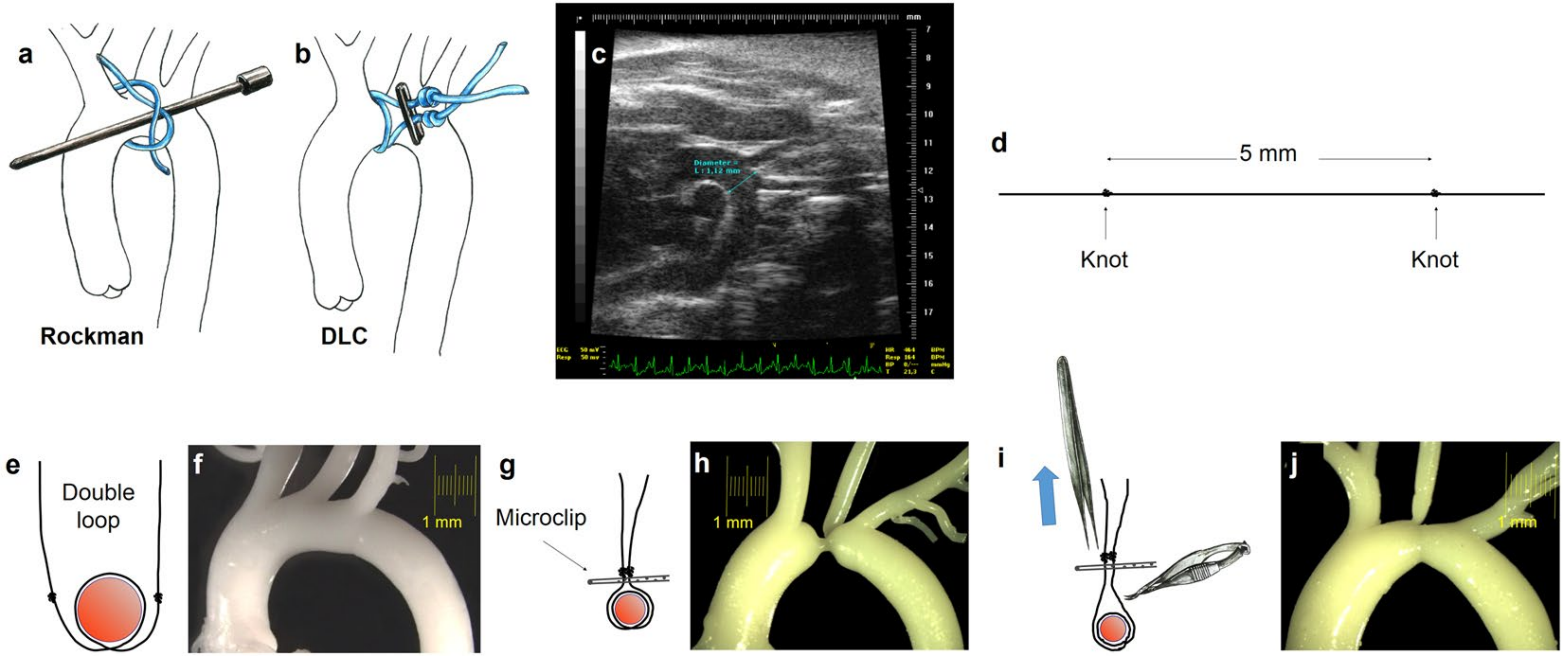
(**a**) Rockman technique. (**b** to **j**) Schematic description of steps for the Double loop-clip technique. (**c**) B-mode echocardiographic image in modified right parasternal view of the thoracic aorta used to measure mid aortic arch luminal diameter, between the innominate trunk and the left carotid artery. This distance was obtained preoperatively and used to calculate the inter-knot span of the suture for the double loop-clip technique and customize the constriction to the mouse´s somatometry. (**d**) The suture was prepared prior to the operation with two knots spread apart a controlled distance (5 mm in the example) intended to define the degree of constriction. (**e**) The suture is looped twice around the mid-aortic arch (**f**), and (**g**) a vascular microclip applied under both knots which produces the preassigned level of stenosis as seen in the silicone cast of the thoracic aorta (**h**). (**i**) During the reoperation for releasing the constriction, pulling from the microclip allows its separation from the aortic wall, which facilitates severance of the suture avoiding the risk of aortic laceration. After release of the constriction the aorta recovers its flow section even though a mild stenosis remains (**h**). DLC: Double loop-clip.

The aortic arch was approached, under spontaneous ventilation, through a midline extrapleural route^5^. A 5 mm midline transverse incision was done over the angle of Louis followed by a 3 mm long longitudinal manubrium sternotomy. The two halves of the split manubrium were spread apart with two stay 5/0 sutures (Prolene^®^, Ethicon Inc.) pulling in ventral-bilateral-caudad direction. The aorta was bluntly dissected with an instrument of our own design, adapted to the surgical field configuration and to the vessel size (Supplementary Figure 2), which allows threading of a 7/0 suture (Prolene^®^, Ethicon Inc.) that is encircled around the mid arch. A wet 27 G cannula was positioned parallel to the mid arch (Fig. 1a). Two sliding and a third locking knots were quickly tied with the suture, adjusted to aorta and cannula, and the latter was gently removed. Additional locking knots prevented loosening of the constriction and its quality was assessed visually. The sternum was closed with 5/0 suture (Vicryl^®^, Ethicon Inc.) and the moist cutaneous edges glued with cyanoacrylate adhesive. Mice received subcutaneous buprenorphine 0.05–0.1 µg/g bid for two days.

#### Double loop-clip technique

TAC was performed with a precalibrated suture customized to the baseline arch luminal diameter (Fig. 1c) and to the desired degree of constriction. The suture was doubly looped around the aorta and a vascular microclip was applied to complete the banding with the predefined circumference (Fig. 1b).

Preparation of the suture loops: The suture for the TAC (7/0 Prolene^®^, Ethicon Inc.) was preset by tying two figure-of-eight knots spread apart a calibrated distance that determined the degree of constriction (Fig. 1d).

The equation for distance calculation between the knots was as follows:

$$Inter-knot\,span=[4\pi \sqrt{[{(\frac{{D}_{L0}}{2})}^{2}\times {(1-St)}^{2}+2h\times (\frac{{D}_{L0}}{2})+{h}^{2}]}]+2Cl$$

where D_L0_ is the baseline luminal aortic diameter (mm), St the fraction of stenosis, h the aortic wall thickness (mm; averaged at 0.06 mm) and Cl the thickness of the vascular microclip (0.4 mm for the one used herein). Should we wish to produce a 70% stenosis in diameter (91% in flow area) in a mouse with a 1.2 mm mid arch aortic diameter, the formula would yield an inter-knot distance of 4.93 mm. The inter-knot span achieved was confirmed with a precision ruler of our own design (Supplemental Fig. 2).

The complete rationale of the formula and macros for calculation of inter-knot span for the obtention of 50, 60 and 70% constrictions in diameter (75, 84 and 91% in flow area) using MicroSoft Excel^®^ software are provided as supplemental material.

Operative technique: Preparation maneuvers, anesthetic technique and surgical approach were similar to those used for RT. The suture with the two knots was encircled twice around the aorta (Fig. 1b,e). The two ends of the suture were gently pulled with a right angled precision forceps (Aesculap Inc., mod. BD333R), avoiding the occlusion of the aorta, so that knots were positioned close to each other and at the same level. The double loop was closed applying a vascular micro clip (Weck Horizon microclips, Teleflex Medical, Research Triangle Park, North Carolina) under both knots (Fig. 1g).

#### Debanding (de-TAC)

Four weeks after TAC the aortic arch was reapproached as described. Immediately after induction of anesthesia, adrenaline (0.2 µg/g) was administered i.m. to prevent vasodilation and cardiodepression. The double loop length of the constricting suture allowed easy separation of the clip from the aortic wall and a safer severance and explantation of the suture and microclip (Fig. 1i). We used for this purpose microscissors with curved serrated jaws (Fine Science Tools, Vannas Tübingen Spring Scissors, # 15071–08).

### Echocardiographic studies

Transthoracic echocardiography was performed under sedation with isoflurane (2.5%), using a Vevo-770^®^ echocardiography unit (VisualSonics, Toronto, Canada) with a 30–45 MHz transducer. The cardiologist performing the studies was blinded to the surgical technique used in the animals. Mid aortic arch luminal diameter was measured preoperatively in modified right parasternal view (Fig. 1c). Transcoarctational pressure gradients were assessed. LV chamber dimensions and wall thicknesses were measured and cardiac mass was estimated. LV ejection fraction (LVEF) and mitral annular plane systolic excursion (MAPSE) were used as surrogates of short axis and longitudinal systolic functions, respectively. The ratio of peak early transmitral flow velocity (E) to peak early myocardial tissue velocity (e′) was used as surrogate of diastolic LV function (E/e′). Supplemental material includes further details.

### Aortic casts

Silicone aortic casts were obtained from sham-operated, four week-TAC, and four week-de-TAC mice (Fig. 1f,h,j). Molding material was injected through the LV apex, filling the arterial tree. After polymerization, the heart and proximal aorta were dissected and soft tissues digested (KOH at 37 °C).

### Histology

Hearts were fixed in paraformaldehyde (3.7% in PBS) and embedded in paraffin. Short axis sections (5 μm, papillary muscles level) (n = 4 mice per experimental condition) were stained using Masson’s trichrome. The fractional area of fibrosis was obtained (ImageJ software) from digital photographs of full LV sections. The minor dimensions of subendocardial cardiomyocytes were measured.

Aortic arch samples were fixed in 3% glutaraldehyde and embedded in Araldite. Transverse sections (1–2 µm) were stained with 0.1% toluidine blue in 0.1% sodium borate and observed under light microscope. Whole aortic arches were fixed in paraformaldehyde, labeled with propidium iodide and studied in toto by confocal microscopy.

### mRNA expression

Total RNA was obtained by TRIzol (Invitrogen) extraction. The mRNA was reverse transcribed using random primers with an RT-PCR kit (Fermentas). The cDNA products were amplified using quantitative PCR (q-PCR) using specific TaqMan assays for TGF-β1, TGF-β2, collagen I (Col I), collagen III (Col III), fibronectin 1 (FN1), and β-myosin heavy chain (β-MHC). The expression was normalized to ribosomal 18 S.

### Statistics

Continuous variables were assessed for normality with the Kolmogorov-Smirnov test. Values are reported as means±S.E.M. Student´s t-test was used to compare means of continuous data. A p value < 0.05 was considered significant. GraphPad Prism 5.01 (GraphPad Software, Inc., La Jolla, CA), Stata 10 (StataCorp LP, College Station, TX) and PASW Statistics 18 (SPSS Inc, Chicago, IL) packages were used. Details in the supplemental material.

## Results

### Aortic luminal diameter does not correlate with somatometric variables

We found no significant correlation between the animal´s body weight or nasoanal length and the aortic arch luminal diameter measured echocardiographically at the baseline study (Supplemental Fig. 3).

### The DLC technique is more efficient than the Rockman technique

**Figure 2:**
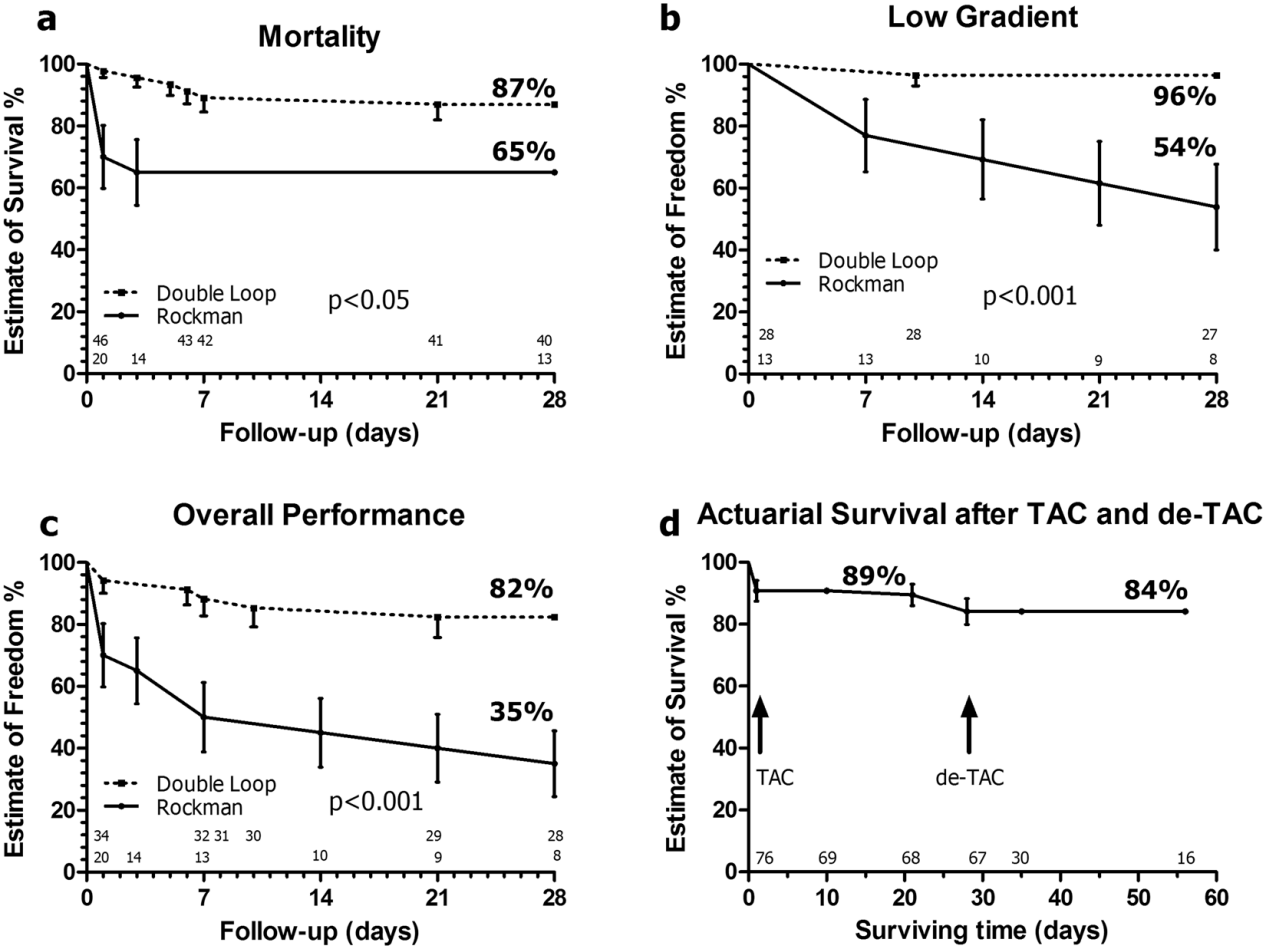
Actuarial analysis of complications after experimental constriction of the mid aortic arch with the RT and DLC techniques. (**a**) Comparison of mortality estimates shows a significant difference favouring the double loop technique and a concentration of early deaths in the Rockman group and relatively constant attrition rate in the DLC cohort. (**b**) Comparison of the probabilities of a low transcoarctational gradient not attributable to LV systolic dysfunction (gradient <30 mm Hg with LVEF>50%). (**c**) Estimates of percent probability of freedom from the combined end-point (death or low gradient) reflect a significant superiority of the double loop technique. (**d**) Actuarial survival, using the DLC technique, for animals operated of TAC and, TAC+de-TAC. Numbers over the X axis represent the animals at risk for each cohort at the corresponding time point. All comparisons with Gehan-Breslow-Wilcoxon test. TAC: Transverse aortic constriction; de-TAC: Release of TAC.

Two competing risks penalize the efficiency of TAC techniques: postoperative mortality and an insufficient constriction (a transcoarctational gradient lower than 30 mm Hg not attributable to LV systolic dysfunction). In our hands, global mortality after TAC was 35% (7/20) with RT and 15% (5/34) with DLP techniques (p NS). Comparison of actuarial mortality estimates (Fig. 2a) shows a significant difference favouring the DLC, and a diverse time course in either cohort with a clustering of deaths during the first three postoperative days in the RT group and a relatively constant attrition rate with the DLC. In the RT group, all deaths were of operative cause. Conversely, among the DLC cohort 3 out of 5 deaths occurred after the first five postoperative days in mice who developed LV systolic dysfunction.

The crude rates of low transcoarctational gradients during the follow-up were 46% (6/13) with RT and 4% (1/28) with DLP (p < 0.01). The actuarial probabilities of a low transcoarctational gradient (Fig. 2b) and freedom from the combined end-point (death or low gradient) (Fig. 2c) reflected a significant superiority of DLC technique. Actuarial estimates of survival, using DLC, are depicted for animals with TAC+de-TAC (Fig. 2d). Survival four weeks post-TAC, including operative mortality, was 89%. Operative mortality of de-TAC was 12% and actuarial survival 4-week post-de-TAC was of 84%.

### The DLC technique provides more consistency in transcoarctational gradients and LV mass increases

**Figure 3:**
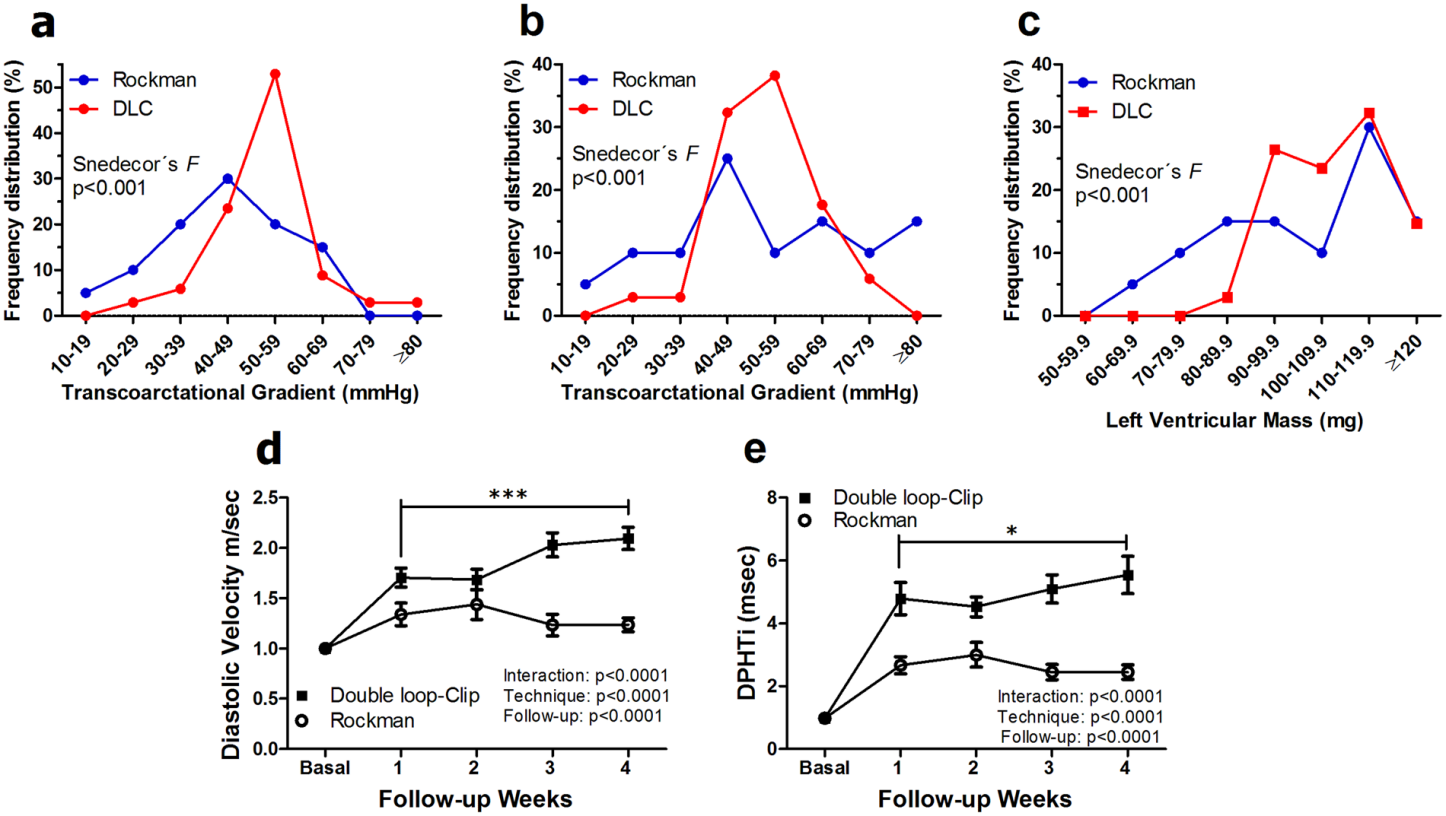
Frequency distribution analysis of postoperative transcoarctational gradients and LV mass developed in mice operated with the RT and DLC techniques. One (**a**) and three (**b**) weeks after TAC, transcoarctational gradients exhibited tightly clustered values in the DLC cohort and a significantly more scattered pattern with RT. (**c**) The distribution of echocardiographically determined LVM three weeks after both procedures showed a similar trend. Comparative analysis of (**d**) transcoarctational diastolic flow velocity and (**e**) diastolic pressure decay index in mice operated with the RT and DLC techniques. The DLC cohort exhibits a more severe impairment to flow as expressed by both parameters and an increasingly severe flow restriction that is reflected by a higher diastolic velocity and DPHTi at four weeks follow-up as compared with one week. DLC: Double loop-clip; DPHTi: Diastolic pressure half-time index. *p < 0.05; ***p < 0.001; both with Student’s *t* test for paired data. All comparisons in the frequency distribution of raw data with Snedecor’s *F* test. Comparisons between time course graphs with repeated measures two-way ANOVA.

Frequency distribution analyses of transcoarctational gradients and LVM after TAC disclosed tightly clustered values in the DLC cohort and a greater scatter with RT (p < 0.001 for the comparison of variances, Snedecor’s *F* test) (Fig. 3a–c). To further assess the severity of TAC we analyzed diastolic velocities and diastolic pressure decays, which disclosed a tighter and increasingly severe flow restriction imposed by DLC as compared with RT. This is reflected by the diverse time course of the diastolic velocities (Fig. 3d) and of the Diastolic Pressure half-time indexes (Fig. 3e). In the DLC group the diastolic flow velocity across the coarctation (p < 0.001) and the diastolic pressure half-time index (p < 0.05) increased significantly from the first to the fourth week after TAC (paired *t* test) (Fig. 3d–e).

### Changes after TAC and de-TAC in LV geometry and function

**Figure 4:**
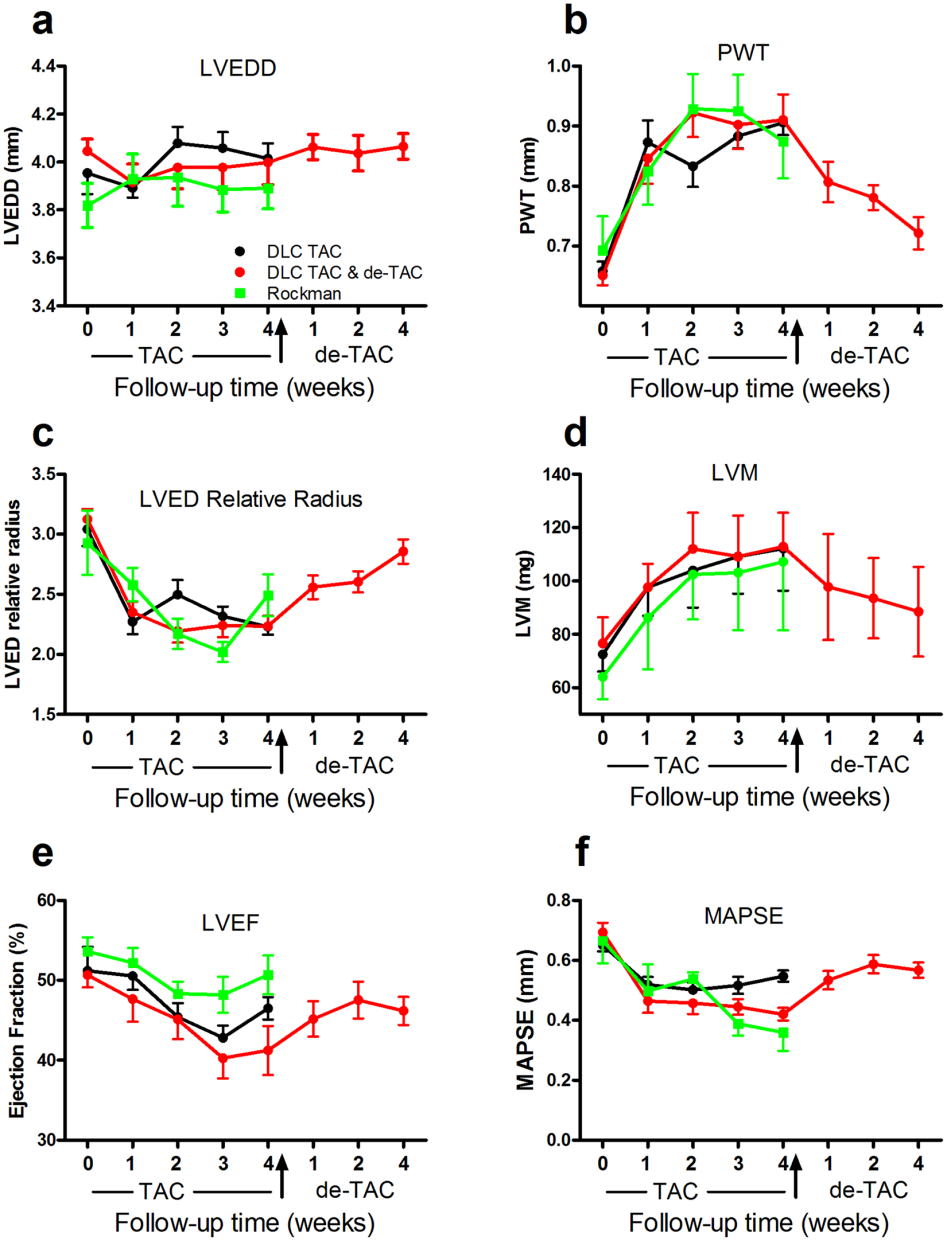
Changes in echocardiographic parameters after TAC and de-TAC with the DLC technique and TAC with the Rockman technique. Error bars represent standard error of the mean except in panel d where they represent standard deviation. Time course of (**a**) LVEDD, (**b**) PWT, (**c**) LVED relative radius, (**d**) LVM, (**e**) LVEF and (**f**) MAPSE. LVEDD: Left ventricular end-diastolic diameter; PWT: Posterior wall thickness; LVM: Left ventricular mass; LVEF: Left ventricular ejection fraction; MAPSE: Mitral annular plane systolic excursion. Measurements presented are the average of three consecutive cardiac cycles. TAC: Transverse aortic constriction. De-TAC: Release of TAC.

Both RT and DLC techniques resulted in LV geometric and functional changes (Fig. 4). PW (and IVS, data not shown) thickened rapidly and significantly, and LVM increased in parallel. LVEDD did not show significant changes after TAC. As a result, the LV modified its shape towards a more concentric geometry. Systolic function was hindered by TAC in both the short (LVEF) and long axes (MAPSE). These changes regressed incompletely after de-TAC.

### TAC promotes cardiomyocyte hypertrophy and interstitial fibrosis which recede after de-TAC

**Figure 5:**
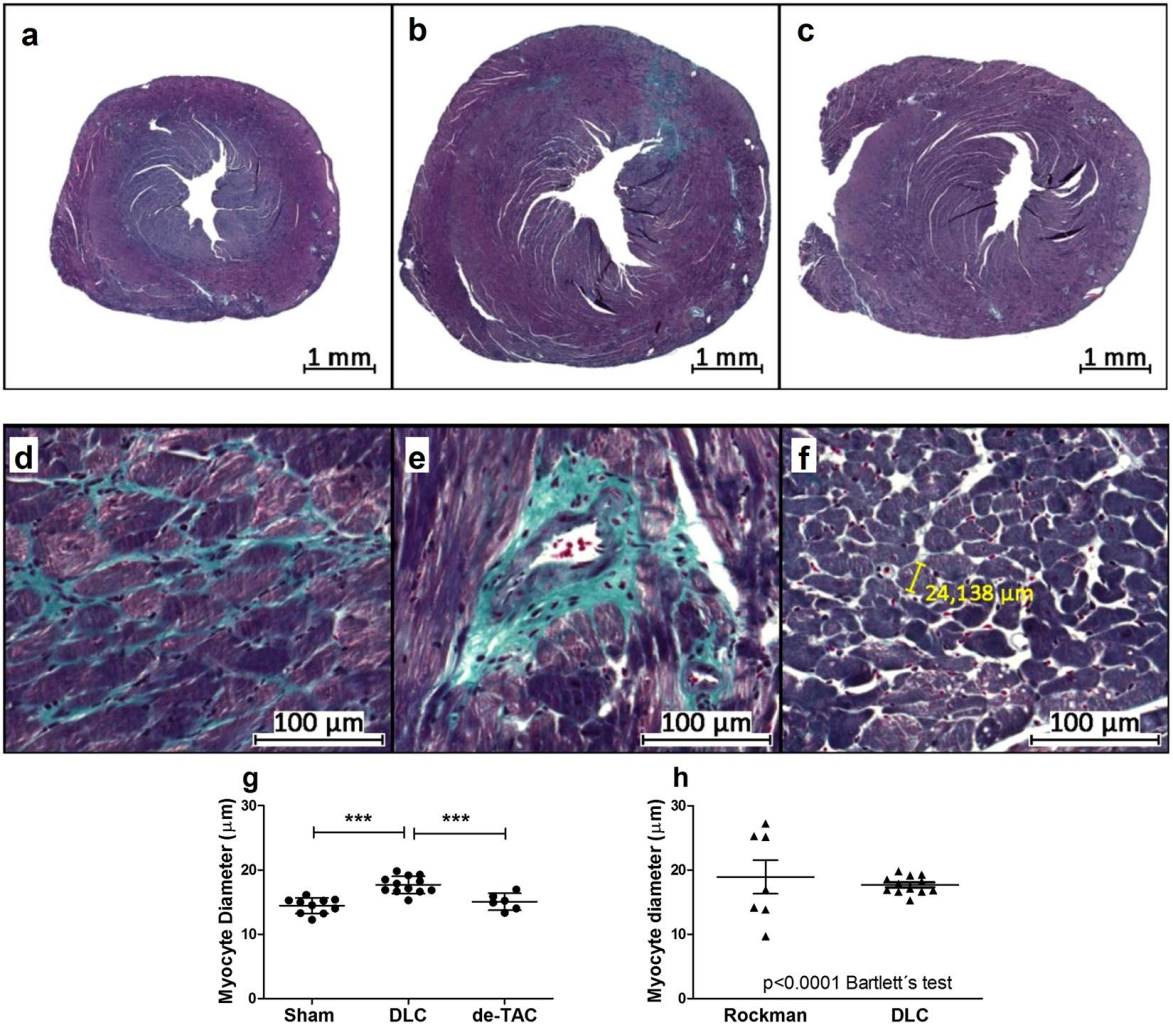
Myocardial histological changes after TAC with the DLC technique. Macrophotographies of LV sections stained with Masson trichrome of (**a**) a control mouse, and mice subjected to (**b**) TAC and (**c**) TAC and de-TAC. Light microscopic studies revealed (**d**) interstitial and (**e**) perivascular fibrosis, and allowed (**f**) morphometric assessment of the cardiomyocytes. (**g**) Cardiomyocyte diameter increased after TAC in the endocardial stratum of the posterior wall. These changes regressed after release of the aortic constriction. (**h**) The dispersion of myocyte diameter values after TAC was significantly greater with Rockman’s technique than with DLC (Bartlett’s test). Comparisons in G with one way ANOVA. **p < 0.01; ***p < 0.001. DLC: Double loop-clip.

Myocardial content of fibrotic tissue increased after TAC, according to the collagen volume fraction, and a significant but incomplete regression occurred after four week de-TAC (Fig. 5a–c). Fibrosis followed mainly a diffuse (Fig. 5d) and perivascular (Fig. 5e) pattern. Cardiomyocyte cross-sectional diameters (Fig. 5f) enlarged after TAC and regressed after de-TAC (Fig. 5g). Comparison of myocyte short-axis diameters in sham and TAC animals with RT and DLC techniques disclosed a significantly wider scatter of values in RT mice, in line with the results of LV mass (Fig. 5h).

### Morphological features of the constricted aortic arch

**Figure 6:**
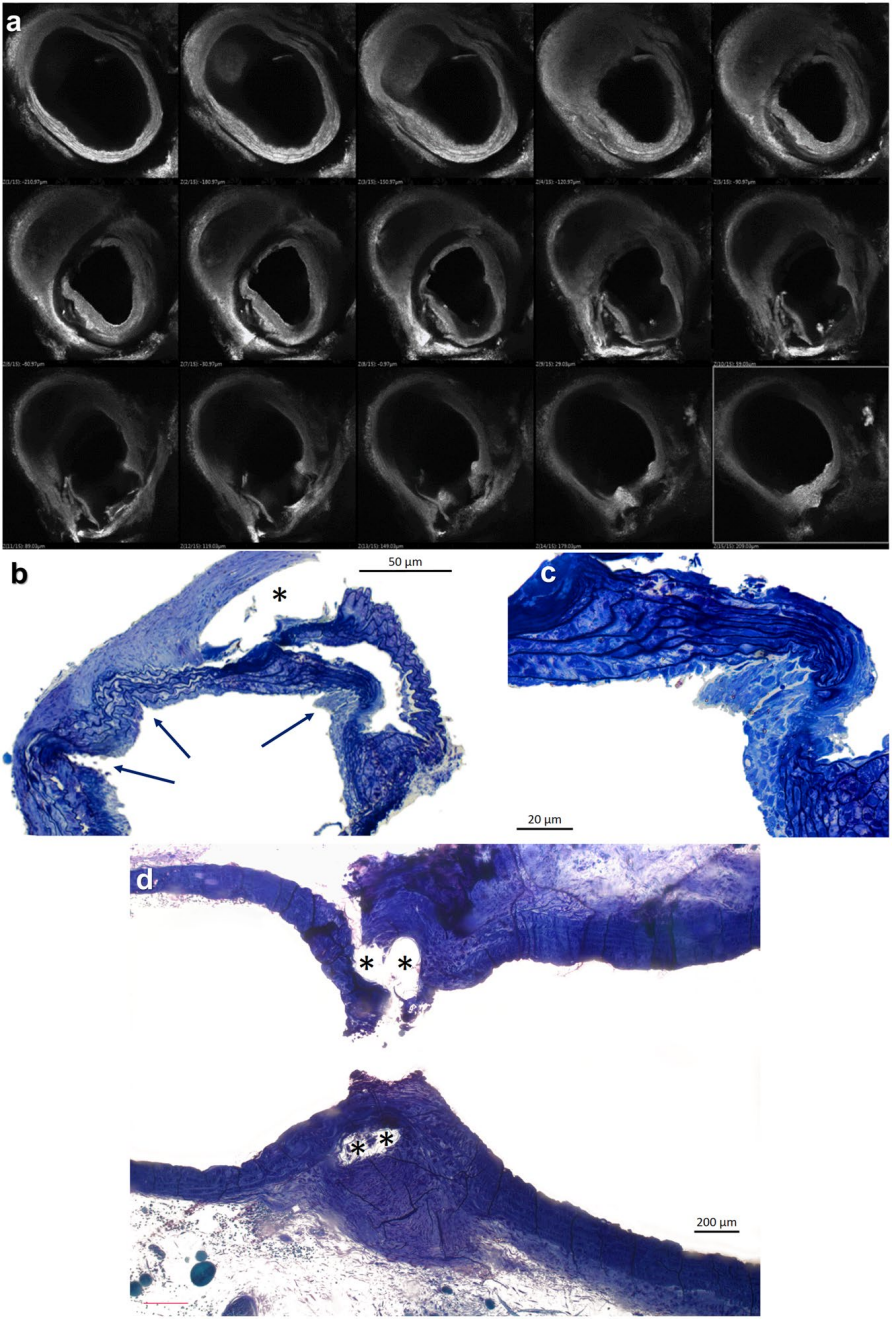
Histological changes in the transverse aortic arch four weeks after TAC with the DLC technique. (**a**) Confocal microscopic images of the murine aortic arch. The transverse sections progress from proximal (upper left) to distal arch (lower right). As the section with the minimal luminal area (middle row, center panel) is approaching, a progressive thickening of the intimal layer is observed. This phenomenon does not appear distally to the constriction. Toluidin blue stained semithin sections of the aortic mid arch in the area of constriction (**b** to **d**). (**b** and **c**) Transverse sections disclose outward folds of the aortic wall in the vicinity of the constriction that (**c**) are partial or totally occupied by cellular elements, whereas the lamellar medial units appear compressed and devoid of smooth muscle cells. Adjacent to the folds the media appears reticulated and thickened with normal cellularity. (**d**) A residual empty channel with a fibrous sheath cover (*) reveals the position of the constricting suture in a longitudinal section.

In animals with 4 week DLC TAC, there was a progressive subintimal thickening composed by an accumulation of smooth muscle cells (Fig. 6a) that increased the degree of lumen restriction in the proximal part of the narrowing. The thickness of this layer increased progressively towards the constriction site. This finding was absent in the early (2 days) phase after TAC and might explain the mild but significant increase in diastolic flow velocity over time in the DLC group after the first week of follow-up (Fig. 3d). Light microscopy studies of the arch revealed the folding of the wall to accommodate the reduced perimeter in the constriction area (Fig. 6b). Vascular wall pleats resulted in compaction of the medial lamellae with a snugly arrangement of the parallel collagen fibers and loss of cells in the bends (Fig. 6c). In the adjacent areas, collagen layers exhibited a thickened reticulated aspect with void formation and preserved cellularity.

### Gene expression data

**Figure 7:**
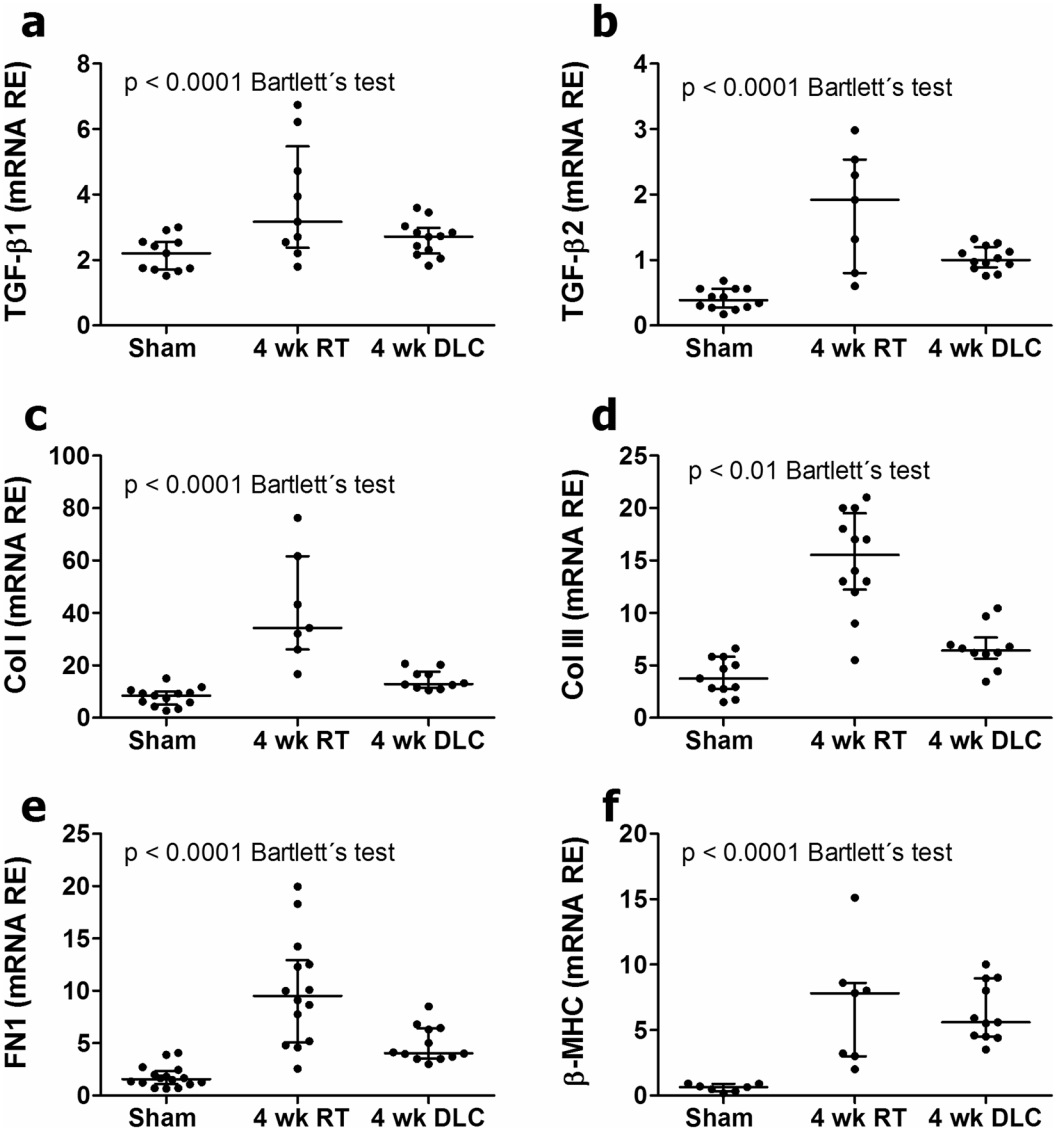
Gene expression levels of remodelling related elements in sham operated mice and mice subjected to TAC with Rockman’s and DLC techniques. Regardless of the mean data, the scatter of relative mRNA values was always significantly greater in the RT than in the DLC cohorts. RE: Relative expression; RT: Rockman’s technique; DLC: Double loop-clip technique; TGF-β: Transforming growth factor beta; Col I: Collagen I-α1; Col III: Collagen III-α1; FN1: Fibronectin1; β-MHC: β-myosin heavy chain.

TAC-mice showed overexpression of cytokines of the TGF-β superfamily (Fig. 7a,b), ECM elements (Fig. 7c–e) and β-MHC (Fig. 7f). Mice undergoing TAC with RT or DLC techniques, exhibited similar trends of rising gene expression, but the scatter of mRNA values was consistently greater in the RT than in the DLC group (Bartlett’s test) (Fig. 7a–f).

## Discussion

Several major issues are responsible of many researchers’ lack of enthusiasm towards Rockman’s TAC pressure overload experimental model:

1. The “*One size fits all”* paradigm. The same reference cannula (27 G) is used to gage the constriction regardless of the sex, weight, body or aortic size of the animals. This leaves a similar luminal area in mice with aortas of different diameters and promotes dispersion of gradients and LV hypertrophy developed. Conversely, our technique is customized to the anatomy of the mouse so as to produce a similar percent reduction in aortic flow area in dissimilar animals.
2. The degree of constriction depends on the tension of the suture after completing the tying maneuver. Hence, with RT the constriction may be more or less severe depending on how tight or loose are the knots and a proportion of animals do not get a significant stenosis immediately after TAC. The reason is easily understood if descriptions of the standard technique^6–8^ are compared to what is taught to any surgical resident on how to get a surgical knot that stays taut after finishing the tying maneuver. Additionally, the complete occlusion of the aortic arch during the tying process forces the operator to spend in this phase as short a time as possible and may explain the swifter but less efficient technique published. Some technical reports are self-explanatory in this respect^6^. This inefficiency of the model is seldom reported, as experiments of mice with a suboptimal degree of TAC or LV hypertrophy in the postoperative controls are simply disregarded^9^. DLC is more controllable, as it does not depend on knot tension and the final constriction is reproducible. Also, the preadjustment of the constricting suture leaves no room for operator-dependent variability of the resulting stenosis. The two knots of the suture lying in parallel and the microclip immediately below them, herald the exact aortic perimeter obtained regardless of the operator’s expertise.
3. Another drawback of RT is the trauma imposed on the vascular wall due to the temporary aortic occlusion at the constriction site. The violent proximal pressure rise, combined with traction and shear stress on the vessel, frequently results in laceration of the aorta and exsanguination of the animal during surgery or at a later date^10^. The alternative to this type of complication is to leave a “not so tight” constriction that penalizes the reproducibility of the model^9^. Additionally, aortic occlusion produces LV overdistention and acute myocardial damage that may either kill the animal or artifact the myocardial features at termination studies. This is particularly relevant for ascending aortic constriction techniques^7,11,12^ where complete aortic occlusion is quickly followed by catastrophic consequences. DLC does not require aortic occlusion, which reduces operative mortality due to aortic rupture and exanguination and precludes acute LV distention.
4. Intraluminal suture migration appears in up to 30% of the animals after RT^9,13^ and is probably facilitated by the tying against bare metal maneuver and by the single loop banding. It promotes a progressive decline of the TAC gradient that constitutes a significant source of error often undetected^13^. This is a complication well known to heart-surgeons after pulmonary artery banding in infants^14,15^ and the sequence of events might be similar in mice subjected to RT-TAC. During surgery, the combined effect of proximal acute overpressure, axial traction and shear stress may induce an incomplete laceration of the aortic wall with contained bleeding and formation of a pseudoaneurism, which will trigger the intraluminal migration of the suture. The phenomenon can be mitigated by placing two separate sutures side-by-side^13^, but elimination of the problem requires an alternative technique, as DLC, which reduces drastically the trauma to the aortic wall.
5. The microsurgical technique of RT is demanding, requires well trained personnel, and associates a variable, and often high, attrition of the animals. This is particularly important when dealing with mutant mice in whom the operative mortality is particularly undesirable^5^. DLC is easier to perform by personnel with basic surgical training and facilitates de-TAC because allows easier separation of the suture from the aorta, preventing wall laceration.
6. RT-TAC surgery mortality in the real-world is difficult to estimate as often is not declared and studies mention only completed experiments. It is influenced by surgical variables like use of mechanical or spontaneous ventilation^5^, type of approach (thoracotomy vs mini-sternotomy), site of the constriction, severity of the stenosis produced (cannula size) and experience of the operator. There is no unified time frame for the definition of mortality and studies refer to this complication as intraoperative, during recovery from anesthesia, within the first 24, 48 or 72 hours, etc. A review of articles reporting this information reveals that early (24 hour) mortality of TAC with a 27 G cannula probably ranges between 6 and 45%^5,6,16^.
7. Aortic de-TAC adds another hurdle to TAC microsurgeries, and implies an extra mortality^9,17^. In our hands, actuarial survival 4 weeks after de-TAC fell an extra 5% with respect to TAC owing to the risk of reoperation.

In summary, the alternative technique proposed is more efficient, generates a customized and consistent aortic arch stenosis and a subsequent homogeneous LV pressure overload. The double loop-clip technique with a premarked suture facilitates a precise, predictable, fully controlled and stable constriction that can be easily performed by laboratory personnel with basic surgical training.

## Electronic supplementary material

Supplementary methods
