# Supplementary material for: Experimental modelling of cardiac pressure overload hypertrophy: Modified technique for precise, reproducible, safe and easy aortic arch banding-debanding in mice
Source: Sci Rep. 2018 Feb 16;8:3167. doi: 10.1038/s41598-018-21548-x (PMC5816612; doi:10.1038/s41598-018-21548-x)
Supplement: Supplementary file 1 — Supplementary methods [file 41598_2018_21548_MOESM1_ESM.pdf]

Experimental modelling of cardiac pressure overload hypertrophy: Modified technique for precise, reproducible, safe and easy aortic arch banding-debanding in mice

David Merino,[2,3] Aritz Gil[1,3], Jenny Gómez[1,3], Luis Ruiz[1,3], Miguel Llano[1,3], Raquel García[2,3], María A Hurlé,[2,3] J. Francisco Nistal,[1,2,3,4]

[1]Hospital Universitario Marqués de Valdecilla, [2]Universidad de Cantabria, [3]Instituto de Investigación Valdecilla (IDIVAL), and [4]Centro de Investigación Biomédica en Red Cardiovascular (CIBERCV), Instituto de Salud Carlos III, Santander, Spain.

Supplemental methods:

Subjects

The experiments were performed in three-month old female mice (C57BL/6J) bred at the University of Cantabria Animal Housing and Experimentation Service (SEEA), housed in a room kept at 22ºC with 12:12 h light/dark cycle, and provided with food and water *ad libitum*. In addition to the experimental groups described in Supplementary Figure 1, sham operated animals were used for cardiomyocyte morphometric (n=10) and gene expression (n=10) studies.

Algorithm for the preparation of the suture loops

Acronyms

A_{L0} = Basal luminal area.
A_{L1} = Luminal area after constriction of 70% in diameter or 91% in area.
A_{loop0} = Basal aortic area (luminal area + parietal area).
A_{loop1} = Aortic area after constriction of 70% in diameter or 91% in area (luminal area + parietal area).
D_{L0} = Basal luminal diameter in mid arch.
D_{L1} = Luminal diameter in mid arch after constriction of 70% in diameter or 91% in area.
h = Mean aortic wall thickness: 0.06 mm or 60 µm
r_{loop1} = Mid-arch aortic radius after constriction of 70% in diameter or 91% in area.

Stepwise development of the equations:

1. $A_{loop1} = A_{L1} + aortic\ wall\ area =$

$$= \left[\left(\frac{D_{L0}}{2}\right)^2 \times \pi \times 0.09\right] + \left\{\left[\left(\frac{D_{L0}}{2}\right) + h\right]^2 \times \pi\right\} - \left[\left(\frac{D_{L0}}{2}\right)^2 \times \pi\right] =$$

$$= \left[\left(\frac{D_{L0}}{2}\right) + h\right]^2 \times \pi - \left[\left(\frac{D_{L0}}{2}\right)^2 \times \pi \times 0.91\right]$$

2. $r_{loop1} = \sqrt{\frac{\left[\left(\frac{D_{L0}}{2}\right)+h\right]^2 \times \pi - \left[\left(\frac{D_{L0}}{2}\right)^2 \times \pi \times 0.91\right]}{\pi}} = \sqrt{\left[\left(\frac{D_{L0}}{2}\right) + h\right]^2 - \left[\left(\frac{D_{L0}}{2}\right)^2 \times 0.91\right]}$

3. $Length\ of\ double\ loop + clip = \left[4\pi\sqrt{\left[\left(\frac{D_{L0}}{2}\right) + 0.06\right]^2 - \left[\left(\frac{D_{L0}}{2}\right)^2 \times 0.91\right]}\right] + 0.8 =$

$$= \left[4\pi\sqrt{\left[\left(\frac{D_{L0}}{2}\right)^2 + 0.12 \times \left(\frac{D_{L0}}{2}\right) + 0.0036\right] - \left[\left(\frac{D_{L0}}{2}\right)^2 \times 0.91\right]}\right] + 0.8 =$$

$$= \left[4\pi\sqrt{\left[\left(\frac{D_{L0}}{2}\right)^2 \times 0.09 + 0.12 \times \left(\frac{D_{L0}}{2}\right) + 0.0036\right]}\right] + 0.8$$

Microsoft Excel™ macros for the calculation of loop lengths

D_{L0} = Baseline luminal diameter in mid arch.

Formula for loop length (mm) with 70% stenosis in diameter
=(4*PI()*(SQRT(((((DL0/2)^2))*0.09)+(0.12*(DL0/2))+0.0036)))+0.8

Formula for loop length (mm) with 60% stenosis in diameter
=(4*PI()*(SQRT(((((DL0/2)^2))*0.16)+(0.12*(DL0/2))+0.0036)))+0.8

Formula for loop length (mm) with 50% stenosis in diameter
=(4*PI()*(SQRT(((((DL0/2)^2))*0.25)+(0.12*(DL0/2))+0.0036)))+0.8

Echocardiographic studies

Postoperative transcoarctational velocities were interrogated using 2D-guided pulsed Doppler at the aortic arch. Gradients were calculated with the modified Bernoulli equation (PG = 4 V^2, where PG is pressure gradient in mmHg and V is velocity in m/seg) from velocity values. However, since transcoarctational velocities do not often reflect fully the severity of the constriction, diastolic velocities and diastolic pressure decays were measured.[i]
Diastolic Velocity (cm/s) was assessed at the end of the T wave of the ECG and Diastolic Pressure half-time Index (ms) or time elapsed for the diastolic pressure gradient to reach half of its peak value. Time variables were normalized to heart rate by Bazett´s method, dividing a given time by the square root of the R-R interval of the ECG.
LV diameters and wall thicknesses were measured from parasternal short axis, 2D-guided, M-mode images at midpapillary level according to international guidelines.[ii] Mitral annular plane systolic excursion (MAPSE) was obtained from four chamber M-mode images at the septal mitral annulus. Parameters of diastolic function were obtained from pulsed wave Doppler analysis of mitral inflow and tissue Doppler interrogation of the posterior wall in the four chamber view. The following parameters were derived from the recordings: heart rate, pressure gradient across the arch constriction, LV end-diastolic (LVEDD) and end-systolic (LVESD) internal dimensions, interventricular septum (IVST) and

posterior wall (PWT) thicknesses, and LV mass (LVM). The relative LV diastolic radius (LVEDr/PWT) was used to assess the concentricity of LV hypertrophy. LV ejection fraction (LVEF) and MAPSE were used as indexes of radial and longitudinal systolic functions, respectively.

LVEF was estimated using the simplified Quinones equation[iii]:

$$LVEF\ (\%) = \frac{LVEDD^2 - LVESD^2}{LVEDD^2} * 100$$

LVM was calculated according to the cube formula[ii]:

$$LVM\ (milligram) = 0.8[1.04(LVEDD + IVST + PWT)^3 - (LVEDD)^3] + 0.6$$

The ratio of peak early transmitral flow velocity (E) to the peak early myocardial tissue velocity (E'), derived from tissue Doppler echocardiography, was used as surrogate of LV filling pressure.

Statistics

Actuarial estimates of postoperative survival, freedom from insufficient constriction and freedom from the combined end-point were calculated using Kaplan-Meier analysis and the resulting curves compared using Gehan-Breslow-Wilcoxon test. Postoperative time-courses of echocardiographic parameters in RT- and DLC-mice were compared by repeated-measures two-way ANOVA. Comparisons of the variances in the frequency distribution of transcoarctational gradients and LVM raw data of RT- and DLC-mice were done using Snedecor´s-*F* test. In sham, TAC and de-TAC in DLC-mice, the cardiomyocyte diameters were compared with one-way ANOVA followed by Newman-Keuls test. Comparisons of the dispersion levels of data was done with Bartlett´s test for equal variances.

Supplementary Figures

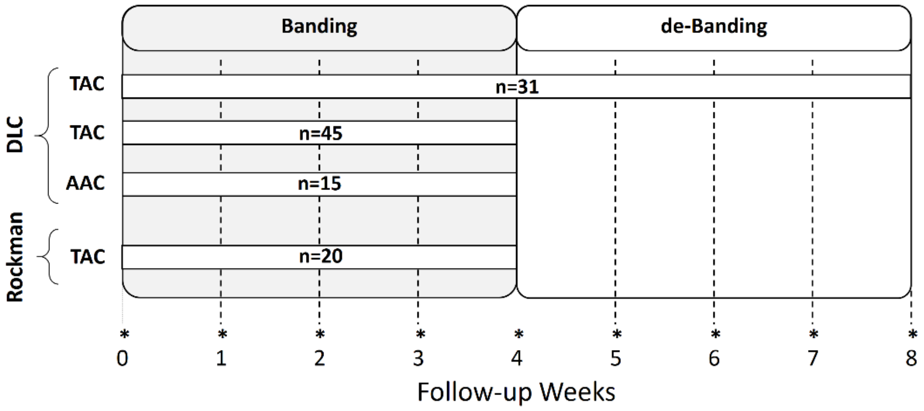

Supplementary Figure 1: Chronogram of the different experimental series and the number of animals in each group for validation of the DLC technique and comparison with RT. Echocardiographic controls were performed at baseline and postoperatively on a weekly basis (*). TAC: Transverse aortic constriction; AAC: Ascending aortic constriction; DLC: Double loop-clip technique.

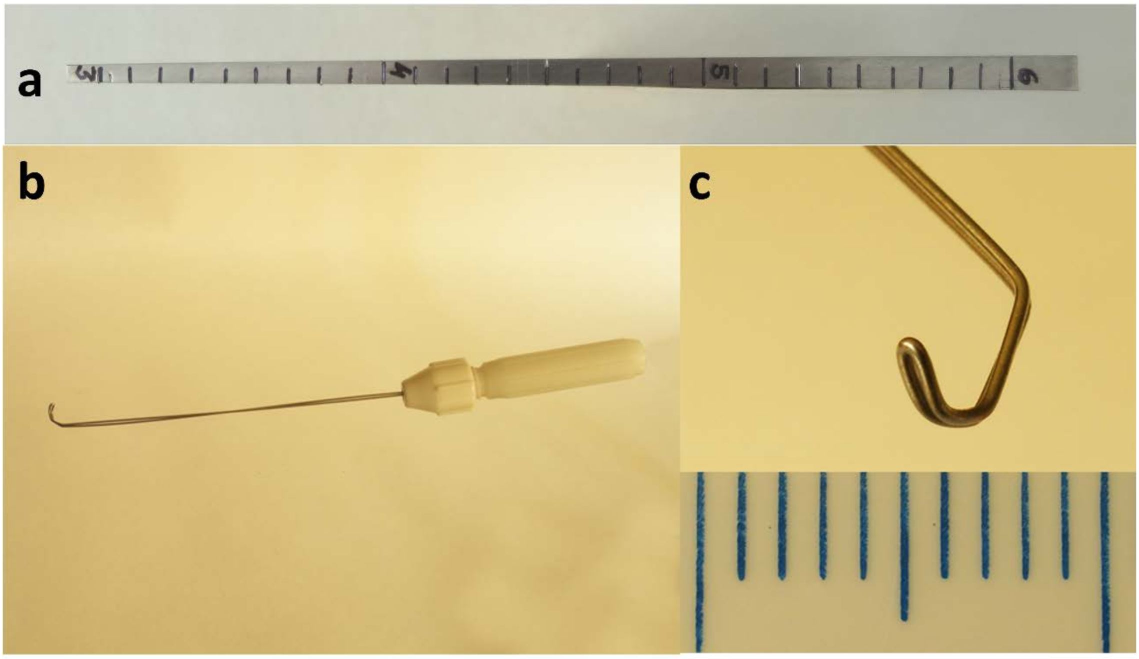

Supplementary Figure 2: Homemade instruments specifically devised for the aortic constriction in mice. 4A) Precision ruler (accuracy <0.1 mm) with convergent edges that allows assessment of the proper concordance between the calculated and the actual inter-knot span of the suture loops prepared for the DLC constriction procedure. 4B and C) General view (B) and detail (C) of blunt dissector of our own design with a double plane curve shape adapted to the geometry of the surgical field and to the size of the murine aortic arch which

allows safe and atraumatic dissection around the vessel and threading of a 7/0 polypropilene suture with the two knots as mentioned above.

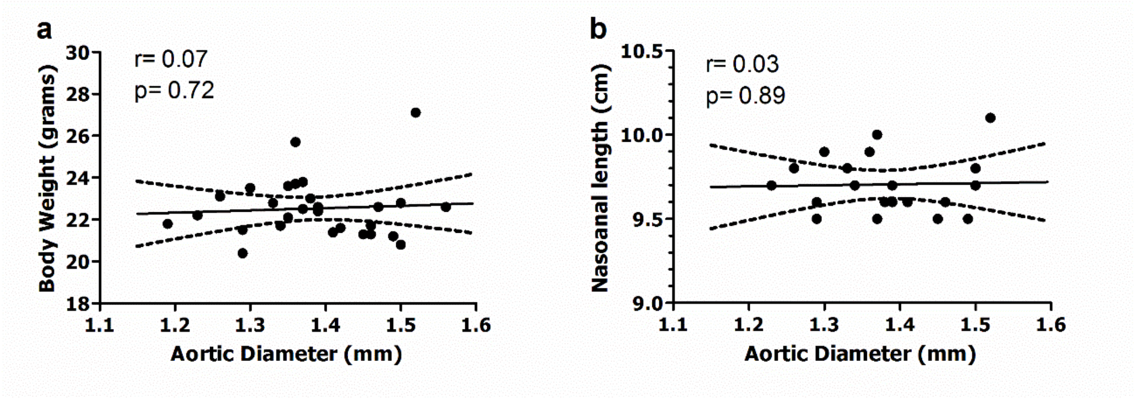

Supplementary Figure 3: Linear regression and correlation analysis of echocardiographically measured mid-aortic arch luminal diameter and body weight (A) or nasoanal length (B) of the mice. There was no significant correlation between the aortic size and either of these variables. Dashed lines delimitate 95% confidence bands.

Supplementary References

[i] Tan JL, Babu-Narayan SV, Henein MY, Mullen M, Li W. Doppler echocardiographic profile and indexes in the evaluation of aortic coarctation in patients before and after stenting. J Am Coll Cardiol. 2005;46:1045-1053.

[ii] Lang RM, Badano LP, Mor-Avi V, Afilalo J, Armstrong A, Ernande L, Flachskampf FA, Foster E, Goldstein SA, Kuznetsova T, Lancellotti P, Muraru D, Picard MH, Rietzschel ER, Rudski L, Spencer KT, Tsang W, Voigt JU. Recommendations for cardiac chamber quantification by echocardiography in adults: an update from the American Society of Echocardiography and the European Association of Cardiovascular Imaging. J Am Soc Echocardiogr. 2015;28:1-39.

[iii] Syed F, Diwan A, Hahn HS. Murine echocardiography: a practical approach for phenotyping genetically manipulated and surgically modeled mice. J Am Soc Echocardiogr. 2005;18:982-990.
